# Comparison of Porcine Tendon to Novel 3D-Printed Silicone Model for Learning Flexor Tendon Repair Techniques

**DOI:** 10.1177/22925503261424896

**Published:** 2026-03-17

**Authors:** Véronique M. Doucet, Abby L. Rentz, Christian J. Petropolis

**Affiliations:** 1 Max Rady College of Medicine, Rady Faculty of Health Sciences, 8664 University of Manitoba, Winnipeg, Canada; 2 Section of Plastic Surgery, Department of Surgery, 8664 University of Manitoba, Winnipeg, Canada

**Keywords:** Simulation, surgery, tendon repair, porcine tendon, 3D printing, chirurgie, impression 3D, réparation des tendons, simulation, tendon de porc

## Abstract

Introduction: Surgical simulation has become an important component of surgical residency. Several animal and synthetic flexor tendon repair simulators have been described, with variable degrees of fidelity. The purpose of this study was to determine the effectiveness of a silicone flexor tendon repair model in comparison to a porcine tendon repair model. **Methods:** A silicone flexor tendon model was created using polypropylene fibres bound in cured silicone to simulate epitenon with the use of a 3D printed mold. Deep flexor tendons were harvested from porcine forelimbs for comparison. Participants tested the models by completing core and epitendinous tendon repairs. Models were evaluated with 5-point Likert Scale questions and a comment section. **Results:** Nine plastic surgery residents and three plastic surgeons participated in the study. Simulation realism was 3.9/5 for the silicone model and 4.6/5 for the porcine model (p = 0.001). Educational utility was 4.6/5 for the silicone model and 4.6/5 for the porcine model (p = 0.546). Overall, the silicone model scored 4.3/5 and the porcine model 4.6/5 (p = 0.078). **Conclusion:** We created a moderate-fidelity tendon repair model that is convenient to use, easily reproducible, and of equal educational utility to a porcine model based on our study results. This model has significant potential for simulation learning in postgraduate surgical education. Further validation is required to confirm its efficacy in postgraduate surgical education and skill transfer to the operating room.

## Résumé

**Introduction :** La simulation chirurgicale est devenue un élément important de la résidence en chirurgie. Il existe plusieurs simulateurs animaux et synthétiques de réparation du tendon fléchisseur, à divers degrés de fidélité. La présente étude visait à déterminer l’efficacité d’un modèle de réparation de tendon fléchisseur en silicone par rapport à un modèle en tendon de porc. **Méthodologie :** Les chercheurs ont créé un modèle de tendon fléchisseur en silicone à l’aide de fibres de polypropylène liées par silicone thermodurci dans un moule en impression 3D pour simuler l’épiténon. Les tendons fléchisseurs profonds ont été prélevés des pattes avant de porc pour des fins de comparaison. Les participants ont fait des réparations du tendon principal et du tendon épitendineux pour mettre les modèles à l’essai. L’évaluation des modèles a été effectuée à partir de questions sur l’échelle de Likert en cinq points et d’une section de commentaires. **Résultats :** Neuf résidents en chirurgie plastique et trois chirurgiens plasticiens ont participé à l’étude. Le réalisme de la simulation était de 3,9 sur 5 pour le modèle en silicone et de 4,6 sur 5 pour le modèle porcin (p=0,001). L’utilité pédagogique était de 4,6 sur 5 pour le modèle de silicone, tout comme pour le modèle porcin (p=0,546). Dans l’ensemble, le modèle de silicone a obtenu un pointage de 4,3 sur 5 et le modèle porcin, de 4,6 sur 5 (p=0,078). **Conclusion :** Les chercheurs ont créé un modèle de réparation du tendon à la fidélité modérée qui est facile à utiliser, facilement reproductible et d’utilité pédagogique égale à celle d’un modèle porcin d’après les résultats de l’étude. Ce modèle a un grand potentiel d’apprentissage par la simulation dans l’enseignement postdoctoral de la chirurgie. Une plus grande validation s’impose pour en confirmer l’efficacité en enseignement postdoctoral de la chirurgie et en transfert des habiletés au bloc opératoire.

## Introduction

The use of surgical simulation in postgraduate medical education aids in the acquisition of foundational surgical skills and in the transfer of these skills to the operating room.^1–4^ The introduction of competency-based education has placed further demand upon surgical simulation devices as tools to demonstrate competency prior to operating on real patients.^5^ The development of high-fidelity (or high-realism) simulation devices is paramount as low-fidelity simulation has been shown to provide only modest, if any, gains in performance.^6,7^

In plastic surgery, performing flexor tendon repairs is a basic yet technically complex skill with a narrow margin of error. A successful repair must be robust enough to withstand early mobilization, while maintaining seamless edges to allow smooth glide through the flexor sheath.^8^ Trainees require practice to develop skill and dexterity prior to attempting independent repair, as a poorly executed repair may result in higher complication rates and considerable functional impairment for patients.^9^

Several animal and synthetic flexor tendon repair simulators have been described in the literature, with variable degrees of fidelity. Animal tendon models (mainly porcine^10^ and bovine^11^) provide a realistic simulation experience where trainees can practice working with real tissue. Porcine flexor tendons are widely utilized due to their similar size, tensile strength, and pulley mechanism compared to human tendons.^12–14^ Animal models have several drawbacks however, including cost, time required for tendon procurement, perishability, strict preservation and disposal practices, and the plausible risk of zoonotic infections.^15^ Synthetic flexor tendon materials described in the literature are numerous and highly variable; they include some of the following materials: Foley catheter,^16^ dental rolls,^17^ micro foam,^18^ liquorice,^19^ bait worm,^20^ glue sticks,^21^ and different formulations of rubber and silicone^22–28^ Hoeyburgh and colleagues^16^ describe a Foley catheter fixed with tape to an anatomic wooden base resembling metacarpal and phalangeal bones. A similar model was developed using a combination rubber-silicone tendon attached to an acrylic model digit, featuring the addition of an FDS and chiasma for added authenticity.^25^ In one study using bait worm, a cohort of trainees showed objective improvement in tendon-repair technique over several simulation sessions followed by a repair on cadavers six months later.^20^ Other lower-fidelity materials such as dental rolls,^17^ micro foam,^18^ liquorice^19^ and glue sticks^21^ have been described without objective measure of their performance in surgical simulation exercises.

Silicone is an inexpensive and accessible material that has been shown to be effective in tendon repair simulation. In a qualitative study by Vinnicombe and colleagues,^23^ three surgeons and seven surgical residents provided qualitative ratings of seven synthetic materials for tendon repair simulation, including urinary catheter, liquorice, bait worm, dental rolls, plastic drinking straw, silicone sealant and the authors’ modification of silicone sealant wrapped in tape to simulate epitenon. Silicone sealant wrapped in surgical tape had a higher score than silicone sealant alone in terms of confidence in the model to facilitate skill performance and its usefulness in surgical training.^23^ Silicone can also be manufactured with varying degrees of hardness and transparency; several studies have utilized transparent silicone so learners can directly visualize the internal placement of their sutures, similar to the glue stick model.^21,22,26^

Recently, three-dimensional (3D) printing has been employed to design and produce customized synthetic tendon simulators. Several groups have described the use of 3D printed silicone tendons to a more anatomically accurate and reproducible synthetic flexor tendon.^26,27^ Furthermore, 3D-printed silicone tendons and simulation mounts have been developed which attempt to recreate the in-vivo environment of flexor tendon repair, complete with metacarpal and phalangeal bones, annular and cruciate pulleys, and added flexor digitorum superficialis tendon.^22,24,26–28^ Determining the validity of these models is an ongoing area of research. In a study by Papavasiliou and colleagues,^24^ FDS and FDP silicone tendons mounted on a 3D printed polylactic acid filament anatomic device. In this study, the performance of junior surgical trainees was evaluated by experienced surgeons and this revealed an improvement in theoretical knowledge and practical skill following use of their 3D printed simulator.^24^ Boyajian and colleagues^26^ demonstrated improved surgical technique in junior learners following a simulation workshop using a 3D printed model, where participants were evaluated by video recording and strength of their repair measured by tensometer. Western et al^27^ objectively studied novice, intermediate and advanced groups using a motion analysis system, to demonstrate the construct validity of a synthetic tendon repair model. Thus far, only one study directly compares porcine and 3D-printed silicone models, in which the 3D-printed model was found to be significantly inferior in tissue feel and realism, and a combined model utilizing porcine tissue on a 3D-printed mount was proposed.^28^

Several materials have been described to simulate human tendon but there is limited evidence of the comparative fidelity and realism of these models. The purpose of this study was to develop an improved 3D-printed flexor tendon repair simulation model and determine the effectiveness of this model in comparison to a porcine flexor tendon model.

## Methods

**Figure 1.**
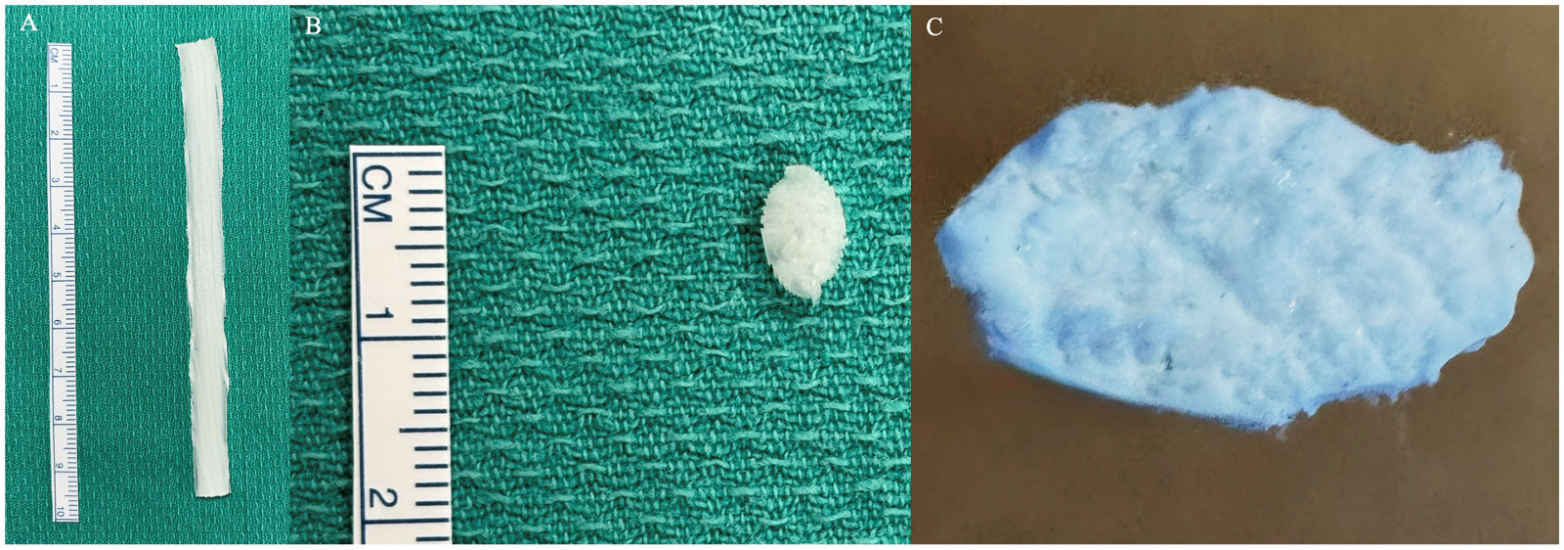
3D-printed flexor tendon model (A), cross-sectional view (B) and detailed cross-sectional view showing densely packed fiber structure (C).

A silicone flexor tendon repair model was created using continuous, longitudinally oriented polypropylene fibers bound in cured silicone with a 3D printed mold developed by the authors using Onshape software (Figure 1). All simulators were printed on a Prusa i3 MK3 printer. The outer silicone layer is intended to simulate the epitenon, while the polypropylene fibers simulate the in vivo alignment of collagen fascicles that compose flexor tendons. The molds were compressed to ensure the fibers made up the majority of the tendon model. Once cured the model was a solid semi-transparent tube measuring 4 × 8 mm in diameter and 150 mm in length. The length of the model can be adjusted by simply cutting it to the desired length. Due to their length, these tendons can accommodate roughly three repair attempts before replacement.

**Figure 2.**
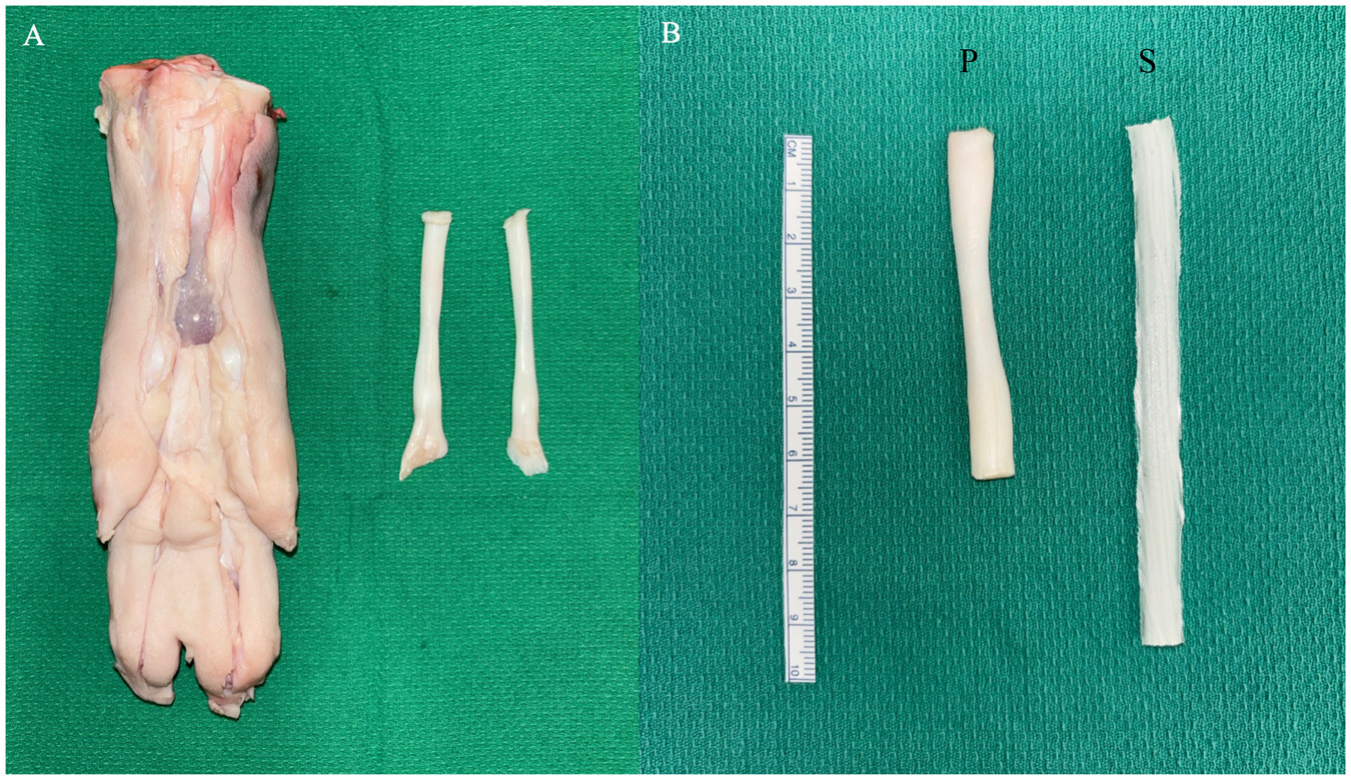
Porcine flexor tendon model harvested from porcine forelimbs (A), and side-by-side comparison of porcine “P” to 3D-printed synthetic “S” model (B).

Porcine forelimbs were collected from commercially slaughtered animals and were refrigerated prior to dissection. Deep flexor tendons were harvested from the deep central rays of the forelimbs by the study authors (Figure 2). Porcine flexor tendons differed in diameter and length due to normal anatomical variation.

**Figure 3.**
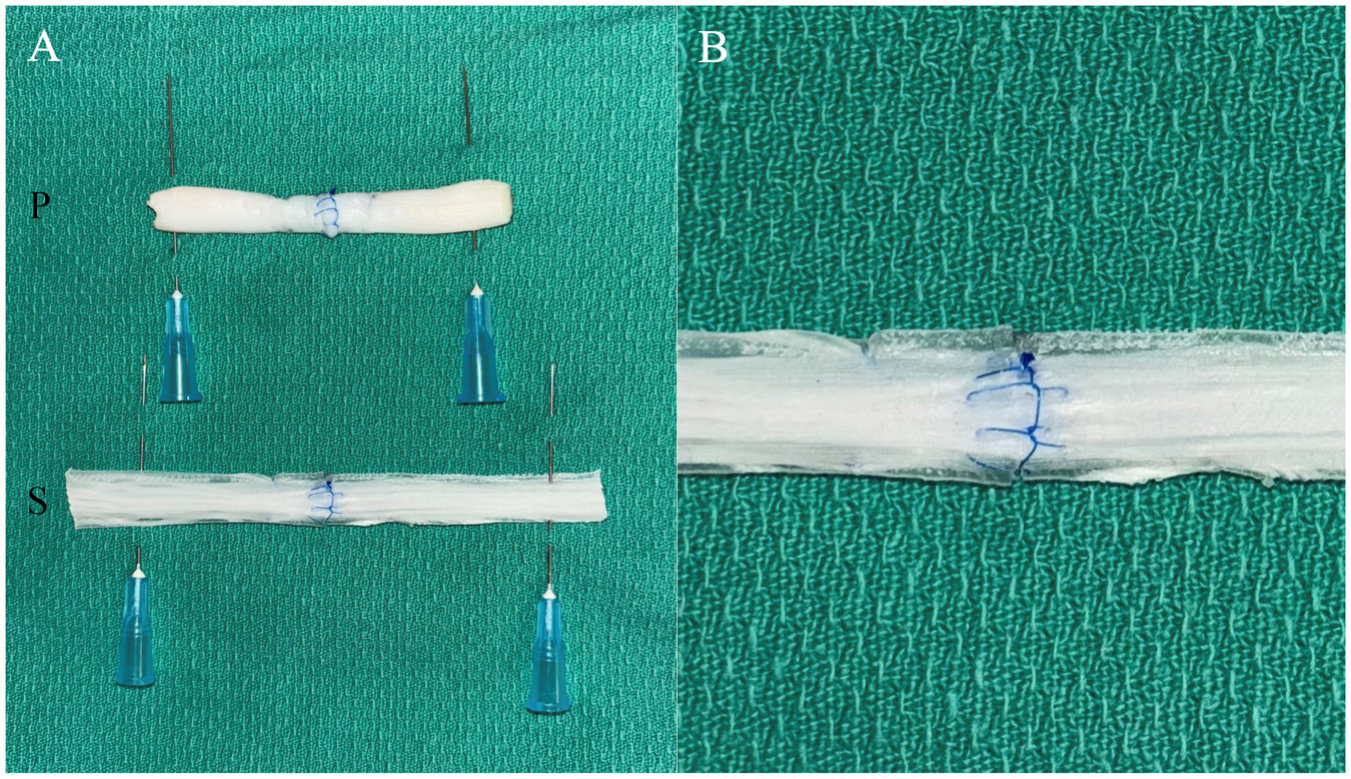
Porcine “P” and 3D-printed silicone “S” flexor tendon repair simulation models following repair with modified kessler core suture and epitendinous suture (A), and close-up of 3D-printed model with longitudinally oriented polypropylene fibers cured in silicone to simulate epitenon (B).

Plastic surgery residents and attending plastic surgeons at the study institution were invited to participate in the study. All voluntary participants were included and evaluated in both tendon repair models. No practice time was allotted with the models before evaluation. Each participant spent approximately 30 min evaluating both models, although no time constraint was given. A transverse cut was made across the middle of the silicone and porcine tendons to simulate a complete tendon laceration injury. Both models were affixed to sterile green towels with anchoring 25-gauge needles to reduce movement during repair. A core tendon repair with a 4-0 non-dissolving braided suture followed by an epitendinous repair with a 5-0 non-dissolving monofilament suture was completed on each model (Figure 3). Participants were instructed to use whichever 4-strand technique they were most comfortable with and to use the same repair technique (core and epitendinous suture repairs) for both models.

All participants completed a de-identified semi-structured questionnaire following model evaluation. Questions to characterize study participants included level of training, number of previous tendon repairs performed, and level of comfort performing tendon repair. Number of previous flexor tendon repairs was graded into 6 categories: no previous repairs, 1 to 10, 11 to 25, 26 to 50, 51 to 100, and more than 100 repairs. Level of comfort doing flexor tendon repairs was graded into 5 categories: not comfortable, somewhat comfortable, comfortable, very comfortable and extremely comfortable. Participants rated the models with 5-point Likert Scale in categories of simulation realism, educational utility and overall reactions, along with a section for comments and open-ended feedback (Table 1).

**Table 1.** Tendon Repair Model Questionnaire.

Simulation Realism	Strongly Agree	Agree	Neutral	Disagree	Strongly Disagree
Model is anatomically accurate					
Tissue feel is realistic					
Resistance of a suture through the tendon is realistic					
Ability to grasp the tendon with forceps is realistic					
Tendon repair strength is realistic					
***Educational utility***	**Strongly Agree**	**Agree**	**Neutral**	**Disagree**	**Strongly Disagree**
Useful for teaching tendon repair techniques					
Useful for improving operative technique					
***Overall reactions***	**Strongly Agree**	**Agree**	**Neutral**	**Disagree**	**Strongly Disagree**
I would recommend this model to other trainees					
This model should be incorporated into our training curriculum					
***Additional questions for trainees only***	**Strongly Agree**	**Agree**	**Neutral**	**Disagree**	**Strongly Disagree**
It would be beneficial to learn and practice tendon repair on this model prior to repairing real tendons					
I would use this model to practice tendon repair independently					

The primary outcome of this study was to determine whether the simulation realism of the silicone model approached or surpassed the standard porcine model, in comparison to participant's experience with human flexor tendon repair. The secondary outcome was to assess the educational utility of the silicone and porcine models. Data were analyzed by calculating means and percentages. A Mann-Whitney U test was used to determine if there was a significant difference between simulation realism, educational utility, and overall rating of tendon repair models. A p value less than 0.05 was considered statistically significant.

## Results

Nine plastic surgery residents and three attending plastic surgeons participated in the study. Plastic surgery residents at all levels of training participated; four junior residents (PGY1 or 2), and five senior residents (PGY3-5). Data collected on the number of previous flexor tendon repairs completed by participants and their level of comfort performing flexor tendon repairs are presented in Table 2.

**Table 2.** Participant Data: Number of Previous Tendon Repairs and Level of Comfort Performing Flexor Tendon Repairs.

Total Number of Participants (n = 12)		Percentage (%)
Number of previous tendon repairs		
None	1	8.3
1 to 10	3	25
11 to 25	–	–
26 to 50	–	–
51 to 100	4	33.3
>100	4	33.3
Participant level of comfort		
Not comfortable	1	8.3
Somewhat comfortable	2	16.7
Comfortable	2	16.7
Very comfortable	2	16.7
Extremely comfortable	5	41.7

**Figure 4.**
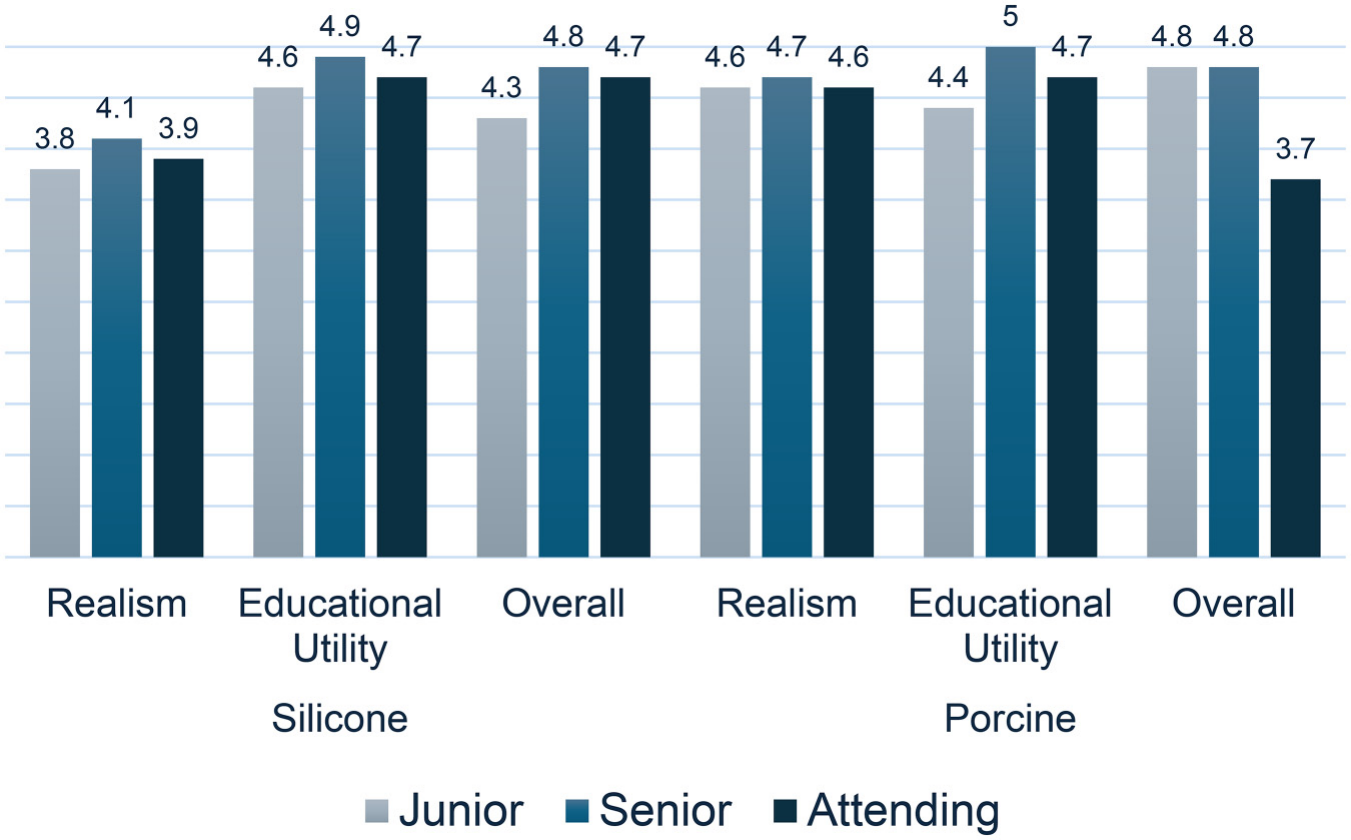
Effect of user experience level on model ratings. Silicone and porcine simulation model ratings were analyzed based on experience level: junior resident (PGY1-2), senior resident (PGY3-5), or attending plastic surgeon.

5-point Likert Scale questionnaire results were broken down into two parts, simulation realism and educational utility (summarized in Table 3). There was statistically significant evidence (p = 0.001) that the porcine model was superior in realism (4.6/5) to the silicone model (3.9/5). There was no statistically significant evidence of difference in educational utility between the porcine model (4.6/5) and silicone model (4.6/5), with p = 0.546. The overall scores of the porcine and silicone models were 4.6/5 and 4.3/5, respectively, which did not show a statistically significant difference (p = 0.078). Analysis of model rating based on experience level (junior resident, senior resident, or attending) is presented in Figure 4. More experienced users (senior residents and attendings) tended to rate both models higher across all domains as compared to junior residents, except porcine overall rating (4.8/5 rating by junior and senior residents, 3.7/5 rating by attendings). However, the sample size within specific groups was insufficient to determine statistical significance.

**Table 3.** Likert Scale Questionnaire Results.

	Silicone Model	Porcine Model	P Value
Simulation realism	3.9/5	4.6/5	0.001*
Educational utility	4.6/5	4.6/5	0.546
Overall	4.3/5	4.6/5	0.078

*Statistically significant result

## Discussion

The development of realistic and accessible simulation models for surgical trainees remains an important area of ongoing study. As with other specialties, surgical simulation in plastic surgery residency training is a constantly evolving practice.^5^ Flexor tendon repair has historically involved training on cadaveric, animal, and artificial simulator models. The results of our study show that although the silicone model scored lower in simulation realism, it was equivalent to the porcine model in educational utility. The silicone model's inferiority in realism is not entirely unexpected as the physical properties of even carefully selected inorganic materials vastly differ from their biologic analogs. Our model differs from previously described 3D printed tendon constructs in the addition of longitudinally oriented fibers bound in silicone to mimic a natural tendon's structure.

The value of a flexor tendon repair simulation models at our institution is reinforced by our finding that the most experienced users rated both models highly in realism and educational utility. Interestingly, the overall rating of the porcine model was much lower than the silicone model in the attending group compared to the resident groups. This may reflect the known inconveniences associated with the use of fresh porcine tissues.

We propose several advantages of the silicone model which make up for its lack in realism, including convenience, low cost, extended shelf life, lack of biohazardous waste, and the ability to easily produce and store large quantities. In addition, the use of 3D printing allows for customization in model shape and size to accommodate different clinical applications and varying levels of surgical training.

3D printing has become a widely available technology with many applications in plastic surgery such as in surgical planning, implant and prosthesis design and production, and information sharing.^29^ We do not expect the cost of labor to be a barrier in the implementation of our model, given that it is a single step process utilizing basic 3D printing equipment, and can produce a batch of hundreds of models simultaneously by one technician. Thus, we anticipate our model could be easily adopted at major medical institutions where 3D printing technology is integrated into clinical practice, and where surgical residency programs would likely be based.

Although porcine simulation models have excellent tissue resemblance to human tissue, they are a limited and expensive resource that requires additional harvesting time and handling techniques. The silicone tendon repair model we presented addresses these issues. Synthetic simulation models help bridge the gap between theoretical knowledge and higher fidelity training models which may be more costly and resource scarce. We believe the current model has sufficient similarity to natural tendon tissue to serve as a useful simulator. This model is ideal for junior residents learning the basic tenets of flexor tendon repair such as various suture techniques, appropriate suture bite distance and depth of suture placement, and to develop hand coordination.

There are several limitations of this study. First, our small sample size of 12 limits the generalizability of our study. Second, the measurement tools used are subjective and not validated; therefore they may not accurately measure the intended variables. Our results also do not provide objective measurement of performance, as this was not the primary aim of the study. Lastly, although 3D printing is an increasingly accessible resource, we recognize it may be technically demanding to unfamiliar users and is unlikely to be available in community settings, which limits the application of our model.

In summary, we created a moderate-fidelity tendon repair model that is inexpensive, convenient, easily reproducible, and of equal educational utility to a porcine model based on our pilot study results. We believe this model has significant potential for simulation learning in postgraduate surgical education. Further validation is required to confirm its efficacy in resident education and skill transfer to the operating room.

## Supplemental Material

**Figure:** Video 1.
